# Comparison of postoperative pain reduction following laser, ultrasonic activation and conventional needle irrigation after root canal treatment – A randomized clinical trial

**DOI:** 10.4317/jced.59898

**Published:** 2023-12-01

**Authors:** Swarna Mathevanan, Nivedhitha-Malli Sureshbabu, Pradeep Solete, Kavalipurapu-Venkata Teja, Jerry Jose

**Affiliations:** 1 Department of Conservative Dentistry and Endodontics, Saveetha Dental College, Saveetha Institute of Medical and Technical Sciences, Saveetha University, Chennai, Tamil Nadu, India

## Abstract

**Background:**

To compare the incidence of postoperative pain and analgesic intake on the administration of passive ultrasonic irrigation (PUI) using IrriSafeTM, laser-activated irrigation (LAI) using Er:Cr: YSGG in comparison to conventional needle irrigation (CNI) in participants diagnosed with symptomatic irreversible pulpitis (SIP).

**Material and Methods:**

In this randomised double-blinded parallel trial, 75 participants requiring root canal treatment in mandibular first molars diagnosed with SIP were enrolled. A 10 cm visual analogue scale (VAS) was used for pain assessment and patients presented with a preoperative pain score of 5 cm and above were only selected. After biomechanical preparation, the final irrigation protocols varied based on the irrigation protocol employed. In CNI, intracanal irrigation was conducted without agitation using a 31G side vented needle. In PUI, irrigant activation was conducted using IrriSafeTM and in LAI, irrigant activation was conducted using a pulsed Er:Cr: YSGG (2940 nm) laser with radial firing tip (RFT - 2). The pain scores and analgesic intake were assessed postoperatively after obturation at 6, 24 and 48 h.

**Results:**

A significant reduction in postoperative pain levels and analgesic intake was seen among all groups at assessed time intervals (*p*<0.05). Overall, mean postoperative pain scores and analgesic intake were CNI>LAI>PUI (*p*<0.05) respectively.

**Conclusions:**

Participants diagnosed with SIP receiving PUI and LAI showed low and comparable levels of postoperative pain scores. Based on the analgesic intake, PUI showed the least analgesic intake when compared to LAI and CNI respectively.

** Key words:**Endodontics, Root canal irrigants, Postoperative Pain, Passive ultrasonic activation, Laser ultrasonic activation.

## Introduction

Currently clinically, conventional needle irrigation (CNI) is shown to be the most commonly employed irrigation with sodium hypochlorite (NaOCl) being the most commonly used biocidal agent based on multiple surveys (1,2). However, conventional irrigation methods fail to deliver intricate parts such as fins and ramifications of the root canal system (3). An element for consideration is the formation of a vapour lock, which can influence the irrigant availability at the apex (4). The small air bubbles which get entrapped during irrigation decrease the irrigant activity.

To overcome these mentioned limitations, emphasis has been placed on various manual or machine-assisted irrigant agitation techniques. Among the plethora of techniques currently administered by clinicians; passive ultrasonic irrigation (PUI), apical negative pressure irrigation and sonic irrigation are the most popular and commonly used (5). A recent irrigation activation modality which has gained recent trend is laser-activated irrigation (LAI) where Er: YAG, Er, Cr: YSGG, Nd: YAG and diode lasers have been studied extensively as irrigant modality for intracanal bacteria reduction and smear removal (6).

Although they were extensively studied, all the above-mentioned lasers might not show their full potential in reducing the bacterial load and complete smear removal. The benefit of this technique depends on the various laser irradiation parameters, type of laser used and physical properties of tissues that ultimately govern the laser tissue interactions. These parameters can cause changes in photomechanical and photoacoustic energy emission where laser pulses with high pulse energy are delivered ultimately causing photothermal changes to the tissues causing a beneficial therapeutic effect at the targeted site (7). Although lasers show a beneficial effect in root canal disinfection there is a scarcity of evidence to suggest a superior laser regimen due to the wide range of characteristics which has to be considered in traditional RCT. In the current study, LAI was done using Er: YSGG 2940 nm laser (Waterlase® iPlus, BIOLASE, USA). Current systematic review (8), although pointed out the heterogeneity among the various parameters employed. The literature evidence has shown the beneficial pain reduction, at 6 hours post-treatment with similar parameters employed in the current study.

Though it is broadly considered that irrigant agitation enables the reduction of postoperative pain and increased healing, the results remain controversial since they vary based on various operative conditions (9,10). Pulpal conditions such as symptomatic irreversible pulpitis are considered to be the most common pulpal pathology with patients exhibiting spontaneous pain often necessitating urgent attention. During this condition, preoperative pain levels have a direct influence on postoperative pain levels in contrast to asymptomatic pulpal conditions (11). Due to all the previously mentioned factors, there is a gap in the literature on the usage of LAI in clinical scenarios; hence we planned to assess the efficacy of LAI and PUI after a single visit to endodontic therapy. Therefore, the current study aimed to evaluate and compare the postoperative pain levels after root canal therapy based on PUI using IrriSafeTM tips (Satelec, Acteon Group, France), Laser activated irrigation and CNI using 31G in teeth diagnosed with symptomatic irreversible pulpitis. The null hypothesis considered was that there is no difference in postoperative pain levels and analgesic intake on using different irrigant activation techniques in patients undergoing single-visit root canal treatment.

## Material and Methods

-Ethical clearance and protocol registration

**Figure 1:**
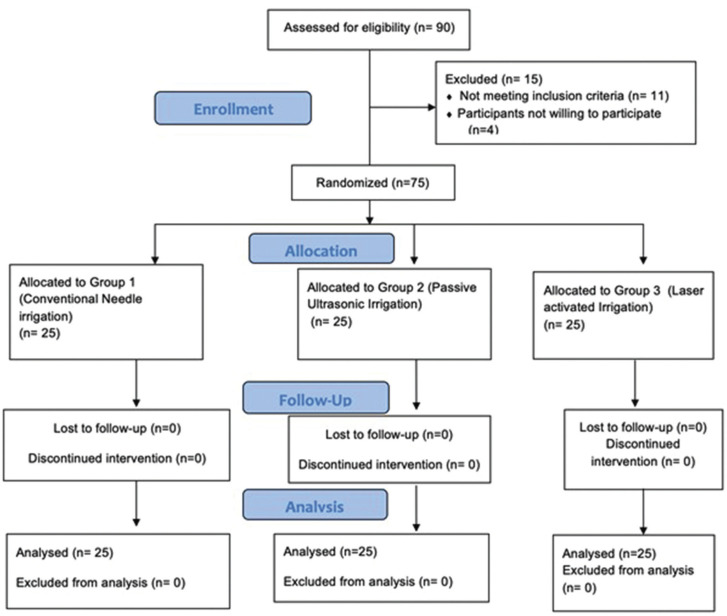
The present study shows the number of participants enrolled and allocated to various irrigation activation group (CONSORT 2010 flow diagram).

This study is a prospective randomized double-blinded and parallel single-centred superiority clinical trial. Before the commencement of the trial, ethical clearance was obtained from the university with an approval number (SRB/SDC/ENDO-1801/20/04) and was conducted according to the code of ethics of the World Medical Association, Declaration of Helsinki. Additionally, the study was registered prospectively in the clinical trial registry - India (CTRI/2021/01/030329). This study is reported using the CONSORT 2010 guideline (Fig. 1).

-Sample Size Determination

As no previous literature assessing similar criteria of postoperative pain reduction using LAI during the time of trial commencement, the sample size was determined based on pilot study results using G*Power version 3.1 for Windows (Henrick Heine-Universität, Düsseldorf, Germany). The estimated power was set at 90%, α = 0.05, f = 0.45. The estimated final sample size was considered to be 66 participants, to compensate for attrition the total sample size was increased by 10% making the sample size a total of 75 participants.

-Eligibility Criteria

a. Inclusion Criteria

Systemically healthy patients (ASA–1) under the age group of 18-65 years with a preoperative pain score ranging more than 5 cm on the VAS scale eliciting severe pain requiring primary root canal treatment in mandibular first molars diagnosed with symptomatic irreversible pulpitis were only considered. Furthermore, radiographic examination using digital periapical radiograph showing the absence of osseous changes, periodontal ligament region widening or periapical region radiolucency with a periapical index (PAI) score of 1 were only considered (12).

b.Exclusion Criteria

Participants, who had consumed analgesics within the last 12 hours or exhibited any systemic illness or allergic reactions to local anesthesia, were excluded. Teeth showing periapical radiolucency and signs of apical periodontitis, crown or root fracture, periapical cyst, resorption defects, calcifications, aberrant anatomy, sinus opening, previous evidence of endodontic treatment and teeth deemed for vital pulp therapies were excluded.

-Setting and Location

The study participants were recruited from the pool of patients who reported for treatment in the postgraduate section of the endodontics department of the college. An initial 90 participants were screened for the possibility of inclusion and the patients were recruited based on prior inclusion/exclusion criteria. The eligible participants were asked to sign an informed consent form that detailed the benefits and risks of the treatment procedure prior to the start of the procedure.

-Randomization, Sequence generation and allocation concealment

The randomization process was carried out by a third person (P.S) who had no participation in conducting the study. Block randomization procedure was followed for the three included groups; CNI, PUI, and LAI. The numbers in each position were randomly allocated. Therefore, for each serial number based on a random number, a group was assigned. A sequentially numbered, opaque, sealed envelope method was implemented for allocation concealment enabling the sequence to be concealed until interventions were assigned. A paper containing a randomized group number was sealed in an opaque envelope containing the respective serial number over it prepared by the prior mentioned individual. Based on the treatment protocol employed, each protocol was mentioned clearly and sealed in allocated envelopes. Participants were assigned their study numbers as they sequentially enrolled in the study. The envelope was opened once the intervention was set. Based on the allocation, the respective treatment based on the group of interventions was conducted.

-Blinding 

This study followed a double-blind design where both the participant and the evaluator did not know the type of intervention being used. As the operator had to conduct irrigant activation using different devices, blinding of the operator was not possible. Once the randomization and allocation concealment were completed, each patient was subjected to a specific treatment protocol and finally assigned to three groups based on the activation protocol followed. A total of 75 participants were included in this study whilst 25 participants were randomly assigned to each group.

-Treatment Procedure

A single operator (S.S.K) carried out all the treatments for all the groups and had 2 years of practice and experience in using the different activation regimens employed in this trial. In the case of proximal caries, before the initiation of treatment, caries excavation was completed and a composite build-up was done to serve as a chamber for irrigant delivery. Local anaesthesia was administered using the inferior alveolar nerve block technique using 1.8 ml of 2% lidocaine with 1:200,000 epinephrine (LOX 2%, Neon Laboratories Ltd, Mumbai, India) before the initiation of treatment. The anaesthetic effect was verified by a lack of response to cold and electric pulp sensibility testing. The tooth was isolated using a rubber dam, and an occlusal reduction of 0.5 - 1 mm was carried out. Access cavity preparation was carried out using access cavity preparation burs (Lexicon Burs, Dentsply Sirona, Switzerland) under a dental operating microscope (OPMI PICO, Carl Zeiss, Oberkochen, Germany). If participants recorded any pain during the procedure, a supplemental intrapulpal injection of local anaesthesia was administered.

The canals were initially scouted using ISO No.8 stainless steel K-file (Mani, Tochigi, Japan). Working length determination was done using an apex locator (Root ZX II, J. Morita, Tokyo, Japan) using an ISO No.10 stainless steel K-file (Mani, Tochigi, Japan) followed by confirmation using a digital periapical radiograph. The glide path preparation was done using continuous rotary instrumentation (ProGlider, Dentsply Sirona, Balligeius, Switzerland) till the recorded working length for each tooth. All the canals were prepared using a continuous rotary system (ProTaper Gold system, Dentsply Maillefer, Balligeius, Switzerland) to 0.5 mm short of apex as suggested based on apex locator readings (Root ZX Mini, J. Moritta Corp. Japan) such that following the manufacturer’s suggested sequence using reduction gear handpiece powered by an electric motor (X-Smart Plus, Dentsply Maillefer, Ballaigues, Switzerland). The apical preparation was done two sizes larger than the initial apical binding file based on a previously published report (13). During the shaping procedure, intermittent irrigation was done using 2 mL of 3% NaOCl (Prime Dental, Thane, India) using a 31G single port side vented needle (Ultradent, South Jordan, UT). Apical patency was maintained throughout the shaping procedure using the previously mentioned ISO No.10 stainless steel K-file between each instrument.

-Irrigation Protocols 

For the CNI group - Following root canal preparation, irrigation was done with conventional irrigation methods using a 31G single port side vented needle (Ultradent, South Jordan, UT). The irrigation was performed using a 27 mm single side-port needle (NaviTip 30G, Ultradent, South Jordan, UT). 3 mL of 3% NaOCl (Prime Dental, Thane, India) was delivered subsequently into each prepared canal such that the needle was not bound and was placed 2 mm short of the working length and moved up and down with 2 mm amplitude for a total time of 30 s. An intermittent flush of 2 mL of physiological saline was carried out after the usage of NaOCl. 3 ml of 17% ethylenediaminetetraacetic acid (EDTA) (Pyrax, Uttarakhand, India) was used as a final rinse for 1 min followed by irrigation using 5 mL of physiological saline to neutralize the effects of EDTA. Excess solution during each phase of irrigation was collected using a high-volume evacuation with a surgical aspiration tip placed on the coronal access cavity preparation.

For PUI group - Following root canal preparation, the irrigants were dispensed into the canal using a 31G single port side vented needle (Ultradent, South Jordan, UT) and the final irrigant activation was done using IRR 25/25 IrriSafeTM file (Satelec, Acteon, France) driven by a P5 piezoelectric ultrasonic unit (Acteon, Mount Laurel, USA) at a power setting of 5 while placing 2 mm short of working length. 3 mL of 3% NaOCl was used for each activation cycle for 30 s for a total of two cycles (9,14) In an instance of curvature in mesial canals, the file was precurved and used for activation. This was followed by an intermittent flush of 5 mL of physiological saline and 3 mL 17% EDTA was activated for 30 s for two cycles followed by a final rinse of 3 mL of physiological saline to neutralize the effects of EDTA.

For the LAI group - Following root canal preparation, the irrigants were activated in a sequence of 5 mL of 3% NaOCl followed by 2 mL of 17% EDTA. The irrigants were dispensed into the prepared canal using a 31G single port side vented needle (Ultradent, South Jordan, UT).Er,Cr:YSGG laser (Waterlase® iPlus, BIOLASE, USA) was used at a wavelength of 2940 nm attached with a radial firing tip (RFT2 Endolase, BIOLASE, USA) with specification 275 μm diameter, 25 mm length, and 0.55 calibration factor with settings of 20 mJ pulse energy, 50 micro sec pulse duration, 10 W power were used, at a frequency of 15 Hz, water and air was turned off during the activation. The laser tip was placed in the prepared root canal system at 2 mm short of working length and 3 mL of 3% NaOCl was dispensed into the canal and activated for 30 seconds for two cycles. An intermittent flush of physiological saline was used and 3 mL of 17% EDTA was used for activation for 30 s for two cycles. A final rinse of 3 mL of physiological saline was used to neutralize the effects of EDTA.

Followed by the activation of irrigants, the canals were dried using corresponding taper paper points (Meta-Biomed, Chungcheongbuk-do, South Korea) and obturated with 6% gutta-percha (Meta-Biomed, Chungcheongbuk-do, South Korea) using AH Plus sealer (Dentsply Sirona, Ballaigues, Switzerland) using cold lateral compaction technique; care was taken so that no sealer extrusion/puff was seen in any of the groups which could potentially influence the results of this study. The coronal seal was done using bonded flowable composite restoration (EverX Posterior, GC Dental, USA). The occlusal was checked and adjusted and the remaining walls and cusps were reduced in height to stabilize tooth structure and a postoperative radiograph was taken to assess the quality of obturation. The time of completion of the procedure and the postoperative pain scores of 6, 24 and 48 h were noted. None of the enrolled patients were prescribed any analgesics and a pain chart was given to record the pain values at 6, 24 and 48 h. In case of severe pain by the patients, they were asked to telephonically call the assistant who was not involved in the treatment procedure and an analgesic (ibuprofen – 400 mg) was prescribed. The total number of Tablets taken was also noted.

-Outcome measures

The primary outcome was to assess the level of postoperative pain at 6, 24 & 48 h using the 10 cm VAS scale which was recorded on a pain diary consisting of the hour and time of assessment. The secondary outcome was to correlate the analgesic intake in patients who reported pain. The participants were not prescribed any medications immediately after the treatment and in the event of severe pain during the follow-up period participants were asked to contact telephonically any of the personnel (K.V.T, J.J) who were not involved in the treatment. The analgesic (Ibuprofen – 400 mg) was prescribed to the participants as a precautionary medication and the quantity of analgesic consumption for each patient was recorded separately.

In an event of excruciating pain, due to the treatment flare up, where the pain cannot be controlled by postoperative prescribed medication, then the participants were asked to report to the clinic and treatment was repeated and were subsequently excluded from the trial. As the experimental protocol was repeated twice in patients where the pain could not be controlled these patients were excluded from the study. After 6, 24 and 48 h, participants were enquired telephonically and asked for their general feeling in the area of the root canal, pain scores and the number of ibuprofen pills that the participants had taken at each follow-up period was recorded on the participant’s chart.

-Statistical Analysis

All the data was imported into the SPSS version 22 (IBM Corporation, USA) statistical analysis software. The normality tests (Shapiro-Wilk test and Kolmogorov-Smirnov) were done to assess the distribution of the data and the data was found to be non-parametric. The Kruskal-Wallis test was chosen for analysing the statistical difference in postoperative pain and analgesic intake among the groups at the assessed time intervals (6,24 and 48 h). One way ANOVA and post hoc Tuckey tests were used for intergroup and intragroup comparisons. A *p* value of less than 0.05 was considered as significance level.

## Results

**Figure 2:**
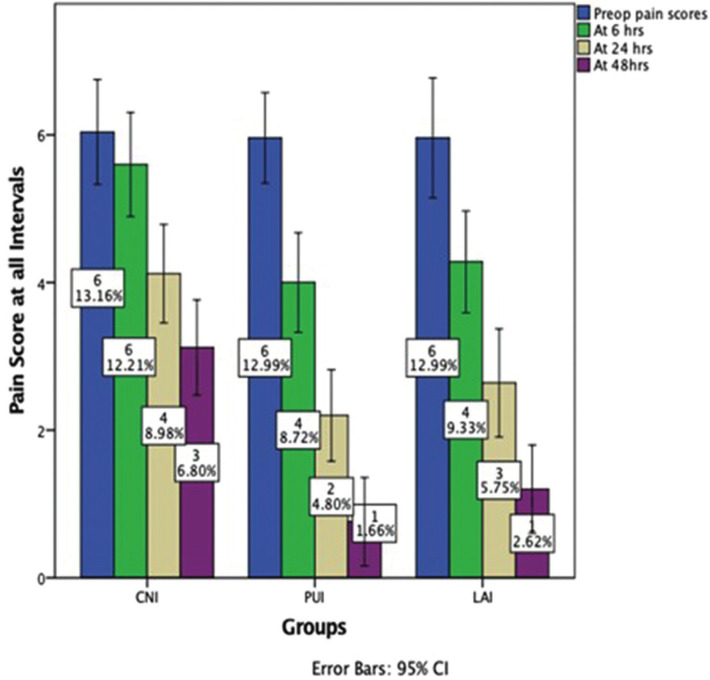
Bar chart showing the pain reduction levels at different point intervals using CNI, PUI and LAI at different assessed time point intervals (preoperative baseline, 6, 24 and 48 hours). Reduction of pain scores was seen in all the groups. It can be seen that PUI showed better reduction of pain compared to LAI and CNI.

From a total of 90 participants who reported pain with mandibular first molars were screened for possible inclusion from the time frame March 2020 to March 2021, 75 participants were enrolled in the current trial. Table 1 denotes the baseline demographic characteristic data of the enrolled participants for this study. All the data were obtained through the pain score sheets and were verified telephonically by an operator (N.M.B) who was not involved in the trial. No loss of follow-up of the participants were seen during the evaluation phase whilst none reported excruciating pain necessitating immediate treatment. The study results showed there was a significant reduction in overall postoperative pain levels at different time intervals assessed (Table 2, Fig. 2). The pairwise comparison showed that PUI and LAI group had a significant reduction of pain scores at all experimental periods when compared to CNI (*p*<0.05), with no significant difference in PUI and LAI in postoperative pain scores at all the assessed time intervals. The consumption of analgesic was assessed and participants who received CNI were subjected to more incidence of analgesic consumption followed by LAI and PUI respectively (*p*<0.05) (Table 3).

**Table 1:**
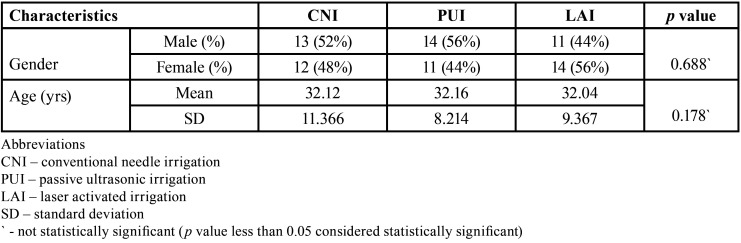
Table showing characteristics of the included participants in the included study. No significant difference was seen between the age and gender.

**Table 2:**
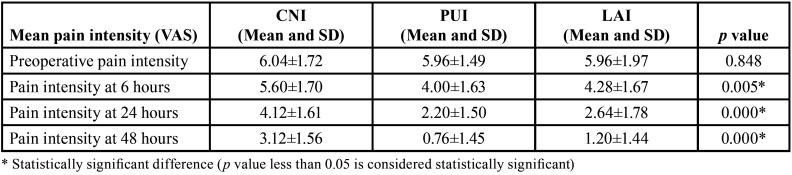
Table showing the mean post operative pain intensity for CNI, PUI and LAI in at different time intervals (preoperative pain, 6, 24 and 48 hours).

**Table 3:**
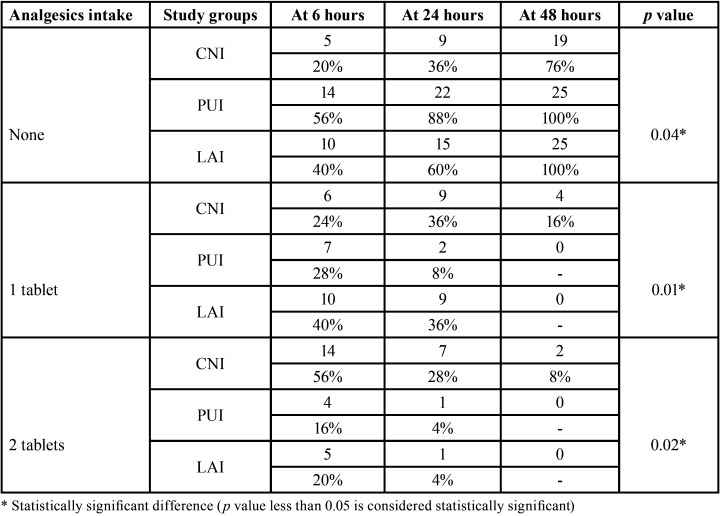
Analgesic intake of patients at different assessed time interval (6, 24 and 48 hours) using irrigation methods using CNI, PUI and LAI.

## Discussion

The present study evaluated the postoperative pain levels of LAI using Er:Cr: YSGG laser, PUI using IrriSafeTM and CNI using a 31G needle and found that CNI resulted in maximum pain score levels compared to other irrigation techniques employed. Current systematic review literature by Nagendrababu *et al*., (15) Justifies that insufficient pulpal debridement with CNI leading to more post-operative pain scores, as compared with the activation. Hence, in the present study as all the cases were completed in the single visit, the effective postoperative pain reduction, depends mainly on thorough disinfection, which could have been compromised in cases where CNI was employed.

To our knowledge, only one randomized controlled trial was conducted proving the superiority of pulsed Er: YAG laser when compared to ultrasonics in participants with asymptomatic teeth undergoing primary root canal treatment (16). The results of this study go by their findings that LAI and PUI exhibited similar levels of postoperative pain reduction despite the different laser protocols employed. Although AAE classification was used in the current study, we have considered patients with moderate to severe baseline pain scores only. Cases deemed for vital pulp therapies were excluded from the study. Cases showed a clear sign of symptomatic irreversible pulpitis with baseline pain scores only considered for the study.

Currently, it has been reported that LAI has superior properties, especially for its bacterial reduction effect and low postoperative pain scores (17). For this trial, mandibular first molars presenting with symptomatic irreversible pulpitis were selected and treated in single-visit endodontic therapy to exclude any confounding variables such as intracanal medication which could have a confounding effect on the results of this study (18). In addition, these teeth are reported to possess the least canal variations specifically in the isthmus region necessitating adequate cleaning and shaping protocol allowing us to specifically select these teeth for inclusion (19). Evidence shows better Irrigant penetration when LAI is used especially in minimal preparations. It is assumed that increasing the apical preparation to two sizes should be enough for better root canal disinfection. A recent report showed pain levels to be less with LAI since they exhibited less apical pressure than CNI during the irrigant activation phase ultimately preventing the extrusion of irrigants from the apex (20). Hence, we could justify the results obtained from our study that LAI showed less postoperative pain when compared to CNI.

The demographic variables of the participants such as age, sex, and preoperative pain levels could play a significant role in influencing the levels of postoperative pain. In this regard, this study was designed in such a way as to avoid confounding variables influencing the results of this study. Further, we selected only participants who were systematically healthy (ASA-1) and who had not consumed any form of analgesics 12 hours before the procedure to avoid the influence of premedication on the underestimation of results (21). To minimize the variations and ensure standardization, in all groups the same treatment protocol was followed except the final irrigation technique to achieve comparable results. During the procedure, optimal care was taken by the clinician to avoid over-instrumentation by accurately determining the working length using a correlation of apex locator and radiograph findings to prevent debris extrusion whilst also obturating without any sealer extrusion/puff preventing variables which can influence the results of this study.

All the procedures for this trial were conducted under rubber dam isolation whilst pre-endodontic build-up was done for proximal caries to ensure adequate disinfection and facilitate irrigant activation. Occlusal reduction was conducted for teeth based on the previous evidence-based by Nguyen *et al*. (22) that though there was a reduction in pain levels 6 days after treatment after occlusion reduction, the results should be taken with caution since they showed no statistical difference on the pain levels from the line of effect in comparison to participants not undergoing occlusal reduction. In the present study, the apical preparation was extended to two sizes. However, the recent study evidence shows the deformation of the apical root canal portions especially in curvatures (23). However, in the current study selection criteria we have excluded the cases with severe canal curvatures. Hence, these effects would be minimal or nil in the present study.

This study showed the highest incidence of postoperative pain in CNI followed by the LAI and PUI group. Regarding analgesic administration, the participants were informed and encouraged to take the pain medication only if there was moderate to severe pain. This was done to avoid over-usage of analgesics that may potentially influence the pain intensity due to treatment protocol in turn affecting the results of our study. Based on the results of our study, the mean analgesic intake was higher in CNI followed by LAI and PUI respectively. Future studies are advised to be carried out extensively on laser and ultrasonic activation in patients undergoing primary and secondary root canal treatment.

The limitations of this study can be the use of a 10 cm VAS horizontal scale which might give over or underestimated results. We recommend future trials to be conducted considering the multitude of factors which can cause pain and using precise pain scales with pain levels. Another limitation of this study is that we did not report the pain scores at 12 h since previous reports had revealed similar levels of postoperative pain scores for participants at a 12-24 h period (24). Another limitation is the consideration of only mandibular first molars diagnosed with symptomatic irreversible pulpitis, though we had taken it for standardization and to avoid any confounding factor, this can be considered to be a limitation since the results achieved could be varied based on different pulpal conduction. The allocation of participants in the current study may be predictable and result in selection bias as the study groups might have been unmasked due to the block randomization process. Hence based on these limitations we recommend future studies to evaluate the tested devices at different periods in participants with different preoperative diagnoses to achieve an accurate outcome and further support the results of this study.

## Conclusions

Based on the limitations of this study, PUI using IrriSafeTM resulted in a reduction of postoperative pain when compared to the LAI using Er, Cr:YSGG and CNI whilst analgesic intake was seen higher in participants who were administered CNI followed by PUI and LNI respectively. PUI exhibited better results compared to LAI with minimal patient discomfort.

## References

[1] Dutner J, Mines P, Anderson A (2012). Irrigation Trends among American Association of Endodontists Members: A Web-based Survey. J Endod.

[2] Savani GM, Sabbah W, Sedgley CM, Whitten B (2014). Current Trends in Endodontic Treatment by General Dental Practitioners: Report of a United States National Survey. J Endod.

[3] Munoz HR, Camacho-Cuadra K (2012). In Vivo Efficacy of Three Different Endodontic Irrigation Systems for Irrigant Delivery to Working Length of Mesial Canals of Mandibular Molars. J Endod.

[4] Tay FR, Gu LS, Schoeffel GJ, Wimmer C, Susin L, Zhang K (2010). Effect of vapor lock on root canal debridement by using a side-vented needle for positive-pressure irrigant delivery. J Endod.

[5] Gu L sha, Kim JR, Ling J, Choi KK, Pashley DH, Tay FR (2009). Review of Contemporary Irrigant Agitation Techniques and Devices. J Endod.

[6] Jurič IB, Anić I (2014). The Use of Lasers in Disinfection and Cleanliness of Root Canals: a Review. Acta Stomatol Croat.

[7] Franzen R, Esteves-Oliveira M, Meister J, Wallerang A, Vanweersch L, Lampert F (2009). Decontamination of deep dentin by means of erbium, chromium:yttrium-scandium-gallium-garnet laser irradiation. Lasers Med Sci.

[8] Elafifi-Ebeid H, Betancourt P, Parada-Avendaño I, Arnabat-Domínguez J (2023). Post-endodontic pain evaluation after different intracanal laser assisted disinfection techniques. A Systematic Review. J Clin Exp Dent.

[9] Căpută PE, Retsas A, Kuijk L, Chávez de Paz LE, Boutsioukis C (2019). Ultrasonic Irrigant Activation during Root Canal Treatment: A Systematic Review. J Endod.

[10] Nagendrababu V, Jayaraman J, Suresh A, Kalyanasundaram S, Neelakantan P (2018). Effectiveness of ultrasonically activated irrigation on root canal disinfection: a systematic review of in vitro studies. Clin Oral Investig.

[11] Sadaf D, Ahmad MZ (2014). Factors Associated with Postoperative Pain in Endodontic Therapy. Int J Biomed Sci IJBS.

[12] Orstavik D, Kerekes K, Eriksen HM (1986). The periapical index: A scoring system for radiographic assessment of apical periodontitis. Dent Traumatol.

[13] Fatima S, Kumar A, Andrabi SMUN, Mishra SK, Tewari RK (2021). Effect of Apical Third Enlargement to Different Preparation Sizes and Tapers on Postoperative Pain and Outcome of Primary Endodontic Treatment: A Prospective Randomized Clinical Trial. J Endod.

[14] Kuah HG, Lui JN, Tseng PS, Chen NN (2009). The effect of EDTA with and without ultrasonics on removal of the smear layer. J Endod.

[15] Decurcio DA, Rossi-Fedele G, Estrela C, Pulikkotil SJ, Nagendrababu V (2019). Machine-assisted Agitation Reduces Postoperative Pain during Root Canal Treatment: A Systematic Review and Meta-analysis from Randomized Clinical Trials. J Endod.

[16] Liapis D, De Bruyne MAA, De Moor RJG, Meire MA (2021). Postoperative pain after ultrasonically and laser-activated irrigation during root canal treatment: a randomized clinical trial. Int Endod J.

[17] Anagnostaki E, Mylona V, Parker S, Lynch E, Grootveld M (2020). Systematic Review on the Role of Lasers in Endodontic Therapy: Valuable Adjunct Treatment?. Dent J.

[18] Izadpanah A, Javaheripour A, Maleki A, Alipour M, Hosseinifard H, Sharifi S (2021). The Comparison of Short-Term Postoperative Pain in Single- versus Multiple-Visit Root Canal Treatment: A Systematic Review and Meta-Analysis Study. Pain Res Manag.

[19] Mohammadzadeh Akhlaghi N, Khalilak Z, Vatanpour M, Mohammadi S, Pirmoradi S, Fazlyab M (2017). Root Canal Anatomy and Morphology of Mandibular First Molars in a Selected Iranian Population: An In Vitro Study. Iran Endod J.

[20] Vidas J, Snjaric D, Braut A, Carija Z, Persic Bukmir R, De Moor RJG (2020). Comparison of apical irrigant solution extrusion among conventional and laser-activated endodontic irrigation. Lasers Med Sci.

[21] Nagendrababu V, Pulikkotil SJ, Jinatongthai P, Veettil SK, Teerawattanapong N, Gutmann JL (2019). Efficacy and Safety of Oral Premedication on Pain after Nonsurgical Root Canal Treatment: A Systematic Review and Network Meta-analysis of Randomized Controlled Trials. J Endod.

[22] Nguyen D, Nagendrababu V, Pulikkotil SJ, Rossi-Fedele G (2020). Effect of occlusal reduction on postendodontic pain: A systematic review and meta-analysis of randomised clinical trials. Aust Endod J.

[23] Shi L, Zhou J, Wan J, Yang Y (2022). Shaping ability of ProTaper Gold and WaveOne Gold nickel-titanium rotary instruments in simulated S-shaped root canals. J Dent Sci.

[24] Smith EA, Marshall JG, Selph SS, Barker DR, Sedgley CM (2017). Nonsteroidal Anti-inflammatory Drugs for Managing Postoperative Endodontic Pain in Patients Who Present with Preoperative Pain: A Systematic Review and Meta-analysis. J Endod.

